# Targeting UGT2B15 and NR1H4 interaction: a novel therapeutic strategy for polycystic ovary syndrome using naftopidil enantiomers

**DOI:** 10.1186/s13048-025-01598-2

**Published:** 2025-01-24

**Authors:** Xiufen Zheng, Zikai Chen, Miao Liang, Liting Zhou, Miaoru Wang, Silin Zhang, Shuyun Zhang, Lei Ma, Wei Yi, Xiawen Liu

**Affiliations:** Key Laboratory of Molecular Target & Clinical Pharmacology and the State Key Laboratory of Respiratory Disease, School of Pharmaceutical Sciences and The Fifth Affiliated Hospital, Guangzhou Medical University, Guangzhou, Guangdong 511436 China

**Keywords:** PCOS, Hyperandrogenism, UGT2B15, DHT, *(R)/(S)*-NAF, NR1H4

## Abstract

**Background:**

Polycystic ovary syndrome (PCOS) is a prevalent endocrine disorder among women of reproductive age. It is characterized by hyperandrogenism, ovulatory dysfunction, and the presence of polycystic ovarian morphology (PCOM) on ultrasound, often accompanied by metabolic disturbances such as insulin resistance and obesity. Current treatments, including oral contraceptives and anti-androgen medications, often yield limited efficacy and undesirable side effects. This study investigates the role of UGT2B15, an essential enzyme for androgen metabolism, in PCOS pathogenesis and its potential as a therapeutic target.

**Methods:**

We used RNA sequencing to examine the effects of UGT2B15 knockdown in KGN cells. To modulate UGT2B15 expression, we employed siRNA and *(R)/(S)*-NAF (naftopidil), a chemical inducer of UGT2B15 identified in our previous studies on a prostate hyperplasia model. The effects of siRNA and *(R)/(S)*-NAF on dihydrotestosterone (DHT) levels, cell apoptosis, and the expression of apoptosis-related proteins in KGN cells were evaluated. In a PCOS mouse model, we assessed the effects of *(R)*-NAF and *(S)*-NAF on serum androgen levels, menstrual cycles, ovarian morphology, and UGT2Bs expression. Additionally, luciferase reporter and ChIP assays were utilized to study UGT2B15 regulation by NR1H4.

**Results:**

Elevated androgens were found to suppress UGT2B15 expression in ovarian granulosa cells, leading to DHT accumulation and apoptosis. *(R)*-NAF and *(S)*-NAF treatments reversed these effects, alleviating PCOS symptoms in mice such as hyperandrogenism, irregular menstrual cycles, and the presence of ovarian cysts. NR1H4 negatively regulated the transcription of UGT2B15 in KGN cells. *(R)*-NAF and *(S)*-NAF disrupted NR1H4 binding to the UGT2B15 promoter without affecting its protein levels, indicating direct interference with its regulation.

**Conclusions:**

UGT2B15 represents a promising target for novel PCOS therapies by modulating androgen metabolism and protecting ovarian granulosa cells from apoptosis. *(R)*-NAF and *(S)*-NAF regulate UGT2B15 by disrupting NR1H4's binding to its promoter, implying potential therapeutic compounds for PCOS treatment.

## Introduction

PCOS, affecting 5–18% of women of childbearing age, is characterized by hyperandrogenism, chronic anovulation, and polycystic ovaries, often with metabolic issues like insulin resistance and obesity [1–3]. Diagnosis follows the 2003 Rotterdam Criteria, requiring at least two of the three features due to clinical variability among patients [2].

Management of PCOS primarily aims to relieve symptoms and mitigate long-term health risks. Common pharmacological treatments include combined oral contraceptives (COCs), anti-androgens, and insulin-sensitizing agents. COCs help with menstrual cycles and hyperandrogenism but raise the risk of venous thromboembolism [4]. Metformin enhances insulin sensitivity and regulates cycles but can cause gastrointestinal issues and vitamin B12 deficiency [5–9]. Anti-androgens are often used with COCs for symptoms like hirsutism and acne [7, 10]. These treatments have limitations and side effects, indicating a need for alternative strategies.

Hyperandrogenism, characterized by clinical symptoms or biochemical markers, is a common but not universal feature of PCOS. Globally, hyperandrogenemia affects 75–90% of PCOS patients, though its prevalence varies across populations [11]. In Chinese women, the prevalence of hyperandrogenemia is comparatively lower but has shown a rising trend in recent years [12]. The pathophysiology of PCOS is complex and involves the dysregulation of androgen production, metabolism, and signaling. Androgens play a key role in regulating ovarian granulosa cell proliferation and apoptosis, processes essential for normal follicular development [13–16]. In the ovarian follicle's theca cells, cholesterol acts as the precursor for androgen synthesis. Upon luteinizing hormone (LH) stimulation, cholesterol undergoes a series of enzymatic reactions, primarily facilitated by cytochrome P450 enzymes, notably CYP17A1. This series of responses includes the conversion of cholesterol to pregnenolone, followed by the synthesis of dehydroepiandrosterone (DHEA) and androstenedione (A4), which are subsequently transformed into Testosterone (T) [17]. T is subsequently transformed into dihydrotestosterone (DHT) by the enzyme 5-alpha-reductase. DHT demonstrates the most potent biological activity with the greatest binding affinity for the androgen receptor (AR) compared to other androgens and plays a vital role in numerous physiological processes [18–20]. The synthesis pathways of testosterone (T) and dihydrotestosterone (DHT) are well-characterized, and current anti-androgen treatments primarily focus on inhibiting androgen synthesis or blocking androgen receptors [21, 22]. However, these therapies, such as spironolactone, are often associated with adverse effects, including hyperkalemia, menstrual irregularities, and fatigue [23–25]. These limitations highlight the need for the discovery of new targets in androgen regulation to improve therapeutic strategies.

In hormone-sensitive tissues, the UGT2B family, particularly UGT2B15, is the primary enzyme mediating the glucuronidation of T and DHT. This process enhances their water solubility and facilitates their excretion, thereby directly regulating local androgen levels and modulating androgen signaling pathways [26, 27]. Consequently, UGT2B15 is implicated in diseases associated with androgen imbalances, and modulating its activity could help regulate androgen levels and improve outcomes in conditions such as benign prostatic hyperplasia (BPH), prostate cancer, and other androgen-dependent diseases [28, 29]. Our previous studies have identified naftopidil (NAF) enantiomers as novel regulators of UGT2B15 in prostate tissues. Specifically, we demonstrated that the NAF enantiomer upregulated the expression and activity of UGT2B15 in both benign prostate hyperplasia (BPH) rat models and human prostate BPH-1 cells [30]. Research on UGT2B15's role in regulating ovarian androgen levels is limited. The potential of UGT2B15 modulators to target hyperandrogenemia and improve PCOS remains unexplored.

This research aimed to investigate how UGT2B15 influences the metabolism of androgens and its possible effects on improving PCOS characteristics in experimental settings. We first evaluated the suppression of androgen buildup by UGT2B15 in KGN cells, indicating its potential role in the development of PCOS. Subsequently, we investigated the protection of UGT2B15 against apoptosis of KGN cells, as evidenced by RNA-seq analysis and subsequent in vitro validation. Furthermore, we provided evidence that *(R)/(S)*-NAF, acting as UGT2B15 inducers, alleviates PCOS-like symptoms in mice, emphasizing the therapeutic promise of targeting UGT2B15 in managing PCOS. Additionally, we discovered that NR1H4 negatively regulates UGT2B15 at the transcriptional level in KGN cells. Our research collectively clarifies the important function of UGT2B15 in regulating androgen metabolism and cell death in ovarian granulosa cells, providing new perspectives on potential treatments for PCOS.

## Materials and method

### Reagents and antibodies

KGN cells were sourced from the American Type Culture Collection (*ATCC*, USA). Fetal bovine serum was obtained from *Gibco* (Carlsbad, USA). Dulbecco's Modified Eagle Medium/Nutrient Mixture F-12 (DMEM/F12) was also procured from *Gibco* (Carlsbad, USA). The UGT2B15 plasmid, small interfering RNA (siRNA), and non-targeting siRNA (YMSW20241231003) were synthesized by *Umine Biotechnology Co., LTD* (Guangzhou, China). *Invitrogen* (Guangzhou, 11,668,019) provided Lipofectamine 2000. Antibodies employed in the trials were anti-UGT2B15 (*Abcam*, ab154864, Cambridge, USA), anti-UGT2Bs (*Affinity Biosciences*, DF7734, Zhenjiang, China), anti-Caspase-10 (*Affinity Biosciences*, AF0122, Zhenjiang, China), anti-Cleaved Caspase-3 (*Cell Signaling Technology*, 9664 T, Danvers, MA), anti-NR1H4 (*Proteintech*, 25055-1-AP, Wuhan, China), anti-Bcl-2 (*Abcam*, ab241548, Cambridge, UK), anti-Bax (*Abcam*, ab270742, Cambridge, UK) and anti-GAPDH (*Affinity Biosciences*, AF7021, Zhenjiang, China). Both *(R)*-NAF and *(S)*-NAF, with enantiomeric excesses greater than 99.5%, were prepared using procedures previously developed by our research group [30]. Letrozole was procured from *Aladdin* (L129473, Shanghai, China). Testosterone (T44450) and dihydrotestosterone (S38542) were obtained from *AcmecBiochemical* (Shanghai, China).

### Cell culture and treatment

KGN cells were cultured in DMEM/F12 medium with 10% fetal bovine serum. KGN cells were transfected with plasmid or siRNA using Lipofectamine 2000 (*Invitrogen*, 11,668,019, USA). Cells were first seeded into six-well plates with a density of 5 × 10^6^ cells per well before transfection. Following a 24-h incubation at 37 °C, cells were transfected with 4 µg/mL plasmid or 50 nM siRNA, combined with Opti-MEM reduced serum medium (Gibco, Carlsbad, USA) and the transfection reagent followed the manufacturer’s protocol for 24 h. Subsequently, cells were treated with full medium, either with or without DHT, *(R)*-NA, and *(S)*-NAF. The exposure time and concentrations of DHT, *(R)*-NAF, and *(S)*-NAF used in the KGN cells were determined based on preliminary experiments that assessed cell viability and pathway responses. Specifically, DHT was applied at a concentration of 500 nM for 24 h, while *(R)*-NAF and *(S)*-NAF were administered at concentrations of 20 µM for 24 h.

### RNA extraction and RNA-seq

RNA was extracted from cells using TRIzol® Reagent, and assessed for quality with a Bioanalyzer (*Agilent*, USA), and only high-quality samples were used for library construction at Shanghai Majorbio (Shanghai, China). 1 µg of RNA was prepared for sequencing with Illumina® Stranded mRNA Prep, using polyA selection, and fragmented with buffer. Following the library construction protocol, cDNA was synthesized with random hexamer primers, followed by end-repair, phosphorylation, and adapter ligation. Libraries were size-selected for 300 bp fragments, amplified by PCR, quantified with Qubit 4.0, and sequenced on NovaSeq X Plus. Reads were trimmed and assessed for quality, then aligned to the reference using HISAT2, and assembled with StringTie to identify differentially expressed genes via transcripts per million reads. Differentially expressed genes (DEGs) were identified using the transcripts per million reads technique and analyzed with DESeq2, designating genes with log2FC ≥ 1.5 and FDR < 0.05 as significant. Functional enrichment analysis, including KEGG, assessed DEGs' significance at *P* < 0.05 against the transcriptome background, using Goatools and Python for pathway analysis.

### Detection of cell apoptosis

Apoptosis of cells was assessed with the Annexin V-FITC Apoptosis Detection Kit from *Beyotime Biotechnology* (C1062L, Shanghai, China). Following 24 h of drug treatment, KGN cells were collected using 2.5% trypsin without EDTA (*Solarbio Science & Technology*, Beijing, China). Afterward, the cells were suspended again in 195 μL of Annexin V-FITC binding buffer. Subsequently, 5 μL of Annexin V-FITC was gently mixed in, then 10 μL of propidium iodide staining solution was added and gently mixed as well. After being kept in the dark at room temperature for 15 min, the cells were examined for apoptosis with a flow cytometer.

### Animal

All animal procedures were approved by Guangzhou Medical University's Animal Care and Use Committee (Approval No. GY2023-482). Thirty female C57BL/6 J mice (3 weeks old, 10–13 g) were obtained from Beijing Vital River Laboratory and housed under SPF conditions.

Due to the small size of mouse ovarian tissues and serum samples, all experiments were conducted with two independent cohorts, each consisting of 6 animals per group. Following one week of flexible feeding, the mice were randomly distributed into five groups (*n* = 6 per group): Vehicle, PCOS, *(R)*-NAF, *(S)*-NAF, and Metformin. Mice in the PCOS, *(R)*-NAF, *(S)*-NAF, and Metformin groups were administered 50 µg/day of letrozole (diluted in 0.1 mL of injectable solution containing 30% PEG300 and 70% saline) via subcutaneous injection. Letrozole treatment was combined with a high-fat diet (Research Diet, D12492, 60% fat) to induce the PCOS phenotype. The Vehicle group received an equivalent volume of the solvent (30% PEG300 + 70% saline) along with a control diet (Research Diet, D12450, 10% fat), as previously described [31]. For the treatment groups, *(R)*-NAF and *(S)*-NAF powders were mixed with 30% PEG400 and 70% saline to a final concentration of 10 mg/mL. Mice in these groups were orally gavaged with the corresponding drug solution. The Metformin group received 200 mg/kg/day of metformin via oral gavage, while the Vehicle and PCOS groups received an equivalent volume of the solvent solution (30% PEG300 + 70% saline) by oral gavage [32]. All treatments were administered over 6 weeks, and body weights were recorded twice weekly. At the end of the treatment period, mice were anesthetized with isoflurane, and blood was collected via cardiac puncture. Tissue samples were preserved at −80 °C for subsequent analysis.

### Monitoring of estrous cycle

From the second week of treatment, vaginal epithelial cells were collected daily at 9:00 AM for cytological smears, assessing estrous cycle stages via Papanicolaou staining over 14 days, distinguishing proestrus, estrus, metestrus, and diestrus stages.

### Histologic staining and follicle counting

Ovarian tissues were fixed in 4% formaldehyde for 24 h, embedded in paraffin, and then sectioned serially at a thickness of 5 µm. Hematoxylin and eosin (H&E) stains were used to stain sections. Follicle counts were performed on every fifth serial section, and the average count per section was determined. The follicles were classified into different groups: primordial follicles with an oocyte surrounded by a single layer of flattened pre-granulosa cells; primary follicles with an oocyte surrounded by a single layer of cuboidal granulosa cells; secondary follicles with two or more layers of cuboidal granulosa cells and no visible antrum; antral follicles with an antral space filled with follicular fluid; and corpus luteum composed of lutein cells [33].

### Immunohistochemistry

Ovarian tissue samples were subjected to immunohistochemical staining with the ABC Staining System. Ovarian sections embedded in paraffin were treated to remove paraffin, rehydrated using xylene and different concentrations of ethanol, and then underwent antigen retrieval in citrate buffer (pH 6.0) at 98 °C for 20 min, before cooling to room temperature. The intrinsic peroxidase activity was inhibited by treating the samples with 0.3% H_2_O_2_ for 10 min. After incubating sections overnight at 4 °C with the UGT2Bs antibody according to the manufacturer's instructions, they were washed three times for 3 min each in 1% PBS. To boost the reaction, secondary antibodies were used for 20 min at room temperature, followed by washing in 1% PBS. The sections were dehydrated using a series of ethanol and then cleared with xylene before being mounted. Positive immunoreactivity was indicated by brown staining in the cytoplasm or nucleus. The H-score, used to quantify the intensity and distribution of staining within tissue sections, was calculated using ImageJ software (*National Institutes of Health*, USA). The H-score for UGT2B15 expression was normalized based on the same tissue area to ensure consistency across samples.

### ELISA assay for cellular DHT, serum hormone levels, and HOMA-IR index

Supernatant samples from KGN cell cultures were collected and centrifuged at 2500 rpm for 5 min at 4 °C. The level of DHT in the supernatant was quantified using an ELISA kit (*FineTest Fine Biotech*, EU2551), following the provided guidelines.

After the drug administration experiment, fasting blood glucose levels in mice were measured using a Roche glucometer after a 12-h fast. Afterward, mice were anesthetized using isoflurane, and blood samples were collected from the orbital venous plexus. After centrifuging at 3000 rpm for 15 min, the serum was separated and stored at −80 °C. The ELISA kits (*FineTest Fine Biotech*) were used to measure the levels of progesterone (EU0398), androstenedione (EU0254), testosterone (EU0400), dihydrotestosterone (EU2551), estradiol (EU0400), and insulin (EM0260) in the serum, following the guidelines provided by the manufacturer. The Homeostatic Model Assessment of Insulin Resistance (HOMA-IR) was determined using the formula: HOMA-IR = fasting glucose (mmol/L) × fasting insulin (mIU/L) / 22.5.

### TUNEL assay

Ovarian tissue samples were sectioned, followed by deparaffinization and rehydration. The sections were incubated with proteinase K solution at 37 °C for 25 min, then treated with permeabilization buffer (*Servicebio*, Hubei, China) for 20 min. According to the TUNEL assay kit protocol (*Roche*, Basel, Switzerland), the tissue was incubated with a mixture of TdT and dUTP at 37 °C for 2 h. Nuclei were counterstained with DAPI (*Beyotime*, Shanghai, China), and fluorescence microscopy (*Nikon*, Tokyo, Japan) was used for observation. The TUNEL staining results were expressed as the positive staining rate, which was normalized based on the tissue area to account for variability in tissue sections.

### Dule-luciferase reporter gene assay

Sequences for UGT2B15 wild-type (WT) and UGT2B15 mutant (MUT, binding site mutation) were created and inserted into the pGL3-basic vector. 293 T cells were seeded in 24-well plates at a density of 2 × 10^4^ cells per well and divided into four treatment groups: pGL3-UGT2B15-WT + pcDNA3.1, pGL3-UGT2B15-WT + pcDNA3.1-NR1H4, pGL3-UGT2B15-MUT + pcDNA3.1, and pGL3-UGT2B15-MUT + pcDNA3.1-NR1H4. Cells were transfected with the corresponding reporter plasmids and the internal control vector pRL-T (Promega, Madison, WI) at a 20:1 ratio (reporter plasmid: control vector) using Lipofectamine™ 2000 (Invitrogen, Carlsbad, CA), following the manufacturer's instructions. Four hours post-transfection, the medium was replaced with DMEM supplemented with 10% FBS, or medium containing 500 nM DHT, 20 μM *(R)*-NAF, or 20 μM *(S)*-NAF as indicated. Luciferase activity was measured with the Dual-Luciferase Reporter Assay System (Promega, USA) after 48 h of transfection.

### Chromatin immunoprecipitation (ChIP) assay

ChIP experiments were performed following the protocol provided by the ChIP kit (*Abcam*, ab500). Formaldehyde was used to fix the cells, followed by sonication of the lysates. The sonicated samples underwent immunoprecipitation using the NR1H4 antibody (*Cell Signaling Technology*, 72,105, USA). Following the purification process, qRT-PCR analysis was conducted. Primers designed for the NR1H4 binding sites of UGT2B15 were used (forward TGTCAAGGGCACCGAACAG, reverse GCCAAGGAGACCAACAAAAGAT).

### Western blot

Total proteins were extracted with RIPA buffer and measured using a BCA assay. Equal protein amounts (30 μg) were separated by 10% SDS-PAGE and transferred to PVDF membranes. Membranes were blocked with 5% milk, incubated overnight with primary antibodies (UGT2B15, UGT2Bs, Caspase-10, Cleaved Caspase-3, NR1H4, GAPDH), and then exposed to HRP-conjugated secondary antibodies. After visualization with chemiluminescence, band intensity was analyzed using ImageJ.

Total proteins were extracted with RIPA buffer and measured using a BCA assay (*Thermo Scientific*, USA). Equal protein amounts of protein (30 μg per sample) were separated by 10% SDS-PAGE and transferred to 0.45 μm PVDF membranes (*Bio-Rad*, USA). Membranes were blocked using 5% non-fat milk (*Cell Signaling Technology*, USA) in TBST at room temperature for 1 h, followed by overnight incubation with the appropriate primary antibodies at 4 °C. In the experiment, antibodies against UGT2B15, UGT2Bs, Caspase-10, Cleaved Caspase-3, NR1H4, and GAPDH were used in different dilutions (anti-UGT2B15, 1:1000; anti-UGT2Bs, 1:1000; anti-Caspase-10, 1:1000; anti-Cleaved Caspase-3, 1:1000; anti-NR1H4, 1:1000; and anti-GAPDH, 1:2000). Afterward, the membranes were washed three times using TBST, the membranes were then exposed to secondary antibodies with HRP-conjugated (*Cell Signaling Technology*, USA), and incubated at room temperature for 2 h. After washed three times with TBST, the membranes were used to visualize protein bands with an enhanced chemiluminescence substrate (*Thermofisher*, USA). Band intensity was analyzed and normalized using ImageJ software.

### RT-PCR quantitative real-time PCR analysis

Total RNA was extracted from KGN cells using TRIzoI reagent (*Life Technologies*, CA). cDNA synthesis was performed utilizing PrimeScript RT Master Mix (*Takara*, China) following the provided guidelines. SYBR Premix Ex Taq (*Takara*, China) was used to perform quantitative real-time PCR (qRT-PCR). The qRT-PCR results were evaluated uusing the comparative gene expression method (ΔΔ cycle threshold). The internal control used was GAPDH. The following primers were used.

**Table:** 

GENE	Forward primer	Reverse primer
siRNA1	GGAGAUAUACCUAAUGUUAdTdT	UAACAUUAGGUAUAUCUCCdTdT
siRNA2	GGAAGAUUCUCUUCUGAAAdTdT	UUUCAGAAGAGAAUCUUCCdTdT
UGT2B4	TGGTGTTTTCTCTGGGGTCG	ACAGCCGAGTATTGAGTCCTA
UGT2B7	CAAACCTGCCAAACCCCTG	GACCCCAGAGAAAACACCACA
UGT2B10	GGTTCTTTGGAGATTTGATGGGA	GCTCTGGTTTTTGGATGACCT
UGT2B15	CTGTAAACCAGCCAAACCCC	ACCCCAGAGAAAACACCACAA
UGT2B17	GCTCTGGGAGTTGTGGAAAGG	GCATTGACAAGAATAGAAGCCGAAGA
UGT2B28	ACCAGGATGGCTCTGAAGTG	ACACCAGCACCTTTCCACAA
GAPDH	CGGATTTGGTCGTATTGG	GCTCCTGGAAGATGGTGAT

### Data analysis

The data were analyzed with SPSS 20.0 statistical software and presented as mean ± standard deviation. Statistical analysis was performed to compare differences between experimental groups. For normally distributed data, one-way ANOVA with Tukey’s post-hoc test was used. When data were non-normally distributed or variances were unequal, the Kruskal–Wallis test was applied instead. Significance levels were represented as follows: ^*^ (*P* < 0.05), ^**^ (*P* < 0.01), ^***^ (*P* < 0.001) denote significant differences between the vehicle group and other experimental groups; ^#^ (*P* < 0.05), ^##^ (*P* < 0.01), ^###^ (*P* < 0.001) indicate significant differences between the model group and other experimental groups; ns indicates no significant difference (*p* > 0.05).

## Results

### UGT2B15 ameliorates androgen accumulation in KGN cells

**Fig. 1:**
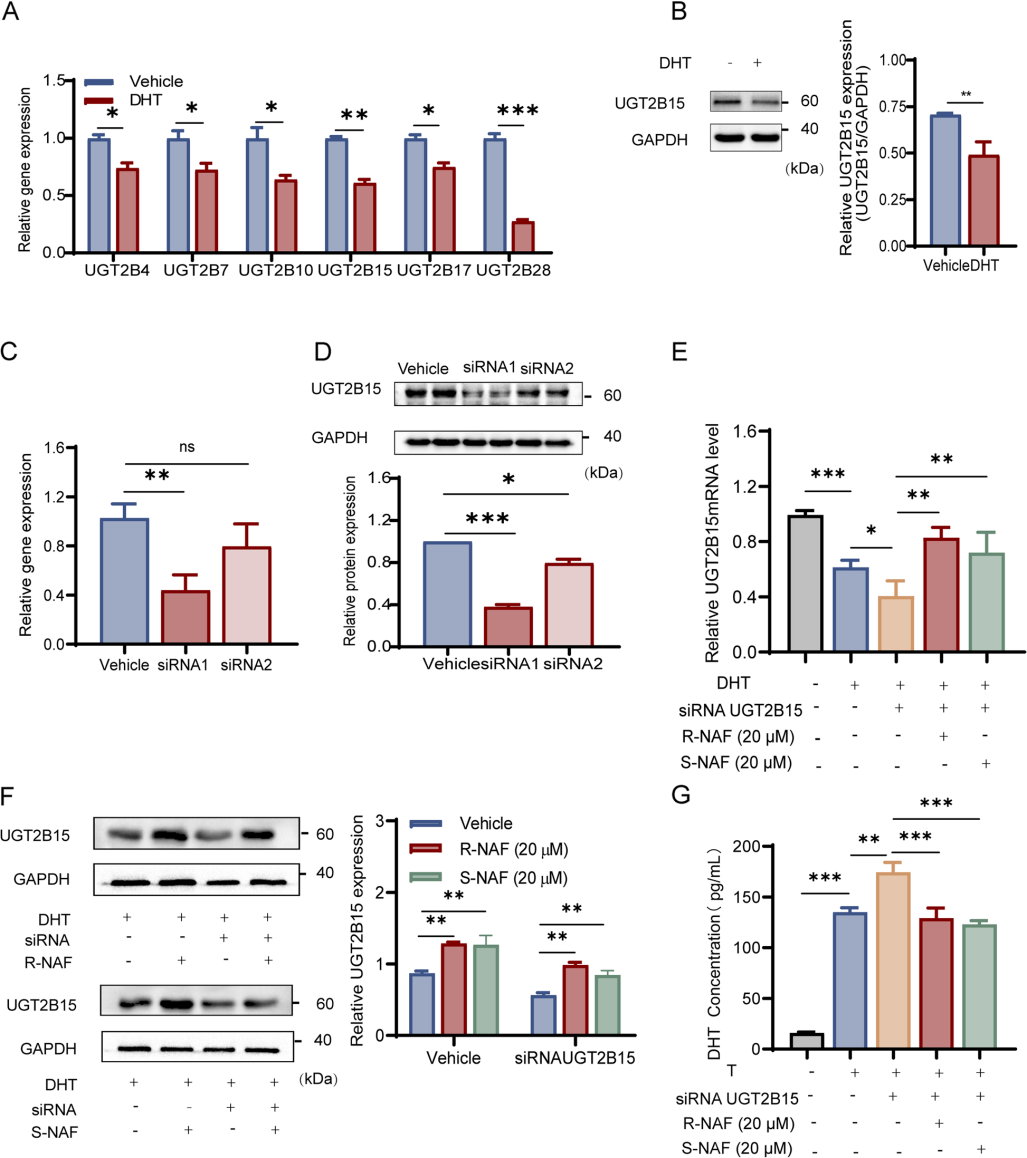
Effect of UGT2B15 on the DHT accumulation in KGN cells. **A** Excessive DHT (500 nM) exposure on gene expression of the UGT2Bs family in KGN cell. **B** Excessive DHT (500 nM) exposure on protein expression of UGT2B15 in KGN cells. **C** Effect of siRNA interferences on UGT2B15 at gene level. **D** Effect of siRNA interferences on UGT2B15 at the protein level. **E** Verify the regulative effects of *(R)*-NAF and *(S)*-NAF on UGT2B15 mRNA expression levels. **F** Verify the regulative effects of *(R)*-NAF and *(S)*-NAF on UGT2B15 protein expression levels. **G** UGT2B15 KD and *(R)/(S)*-NAF played opposite roles in the regulation of DHT accumulation induced by excessive T (500 nM) exposure. Results of all qPCR and Western blot were standardized based on GAPDH levels and are expressed in comparison to the vehicle group. Values are expressed as mean ± SD (*n* = 3–5 per group). *P* values were determined by one-way ANOVA with Tukey's multiple comparison post-hoc test. ^*^P < 0.05, ^**^P < 0.01, ^***^P < 0.001, ns means no significant difference

Granulosa cells are pivotal in determining follicular fate by providing essential molecules for growth and maintenance and undergoing apoptosis, leading to follicular atresia [34]. Excessive androgen production is a defining characteristic of PCOS. To create a hyperandrogenic PCOS cell model, we treated KGN cells with excessive DHT (500 nM) as in the previous study [35]. As illustrated in Fig. 1A, the UGT2B gene family, including UGT2B4, UGT2B7, UGT2B10, UGT2B15, UGT2B17, and UGT2B28, showed significant expression reduction due to DHT exposure. UGT2B15 exhibited the most significant reduction at the protein level (Fig. 1B).

Non-targeting siRNA was used as the negative control to assess the specific effects of target gene knockdown and was designated as the Vehicle group. UGT2B15 was modulated using siRNA, confirming knockdown at mRNA and protein levels. *(R)*-NAF and *(S)*-NAF were used to enhance UGT2B15 expression, confirming their effects on DHT levels (Fig. 1C-F). siRNA1 was selected for subsequent experiments. Additionally, *(R)*-NAF and *(S)*-NAF were utilized based on our previous findings showing their effectiveness both in laboratory settings and in living organisms [30]. UGT2B15 knockdown led to a notable rise in DHT levels in KGN cells, which was then counteracted by *(R)*-NAF and *(S)*-NAF resulting in decreased DHT accumulation (Fig. 1G).

In summary, UGT2B15 regulates DHT levels in KGN cells, reducing DHT accumulation from excess androgen, while *(R)*-NAF and *(S)*-NAF stimulate UGT2B15 and further prevent DHT buildup.

### UGT2B15 regulated the KGN cell apoptosis

**Fig. 2:**
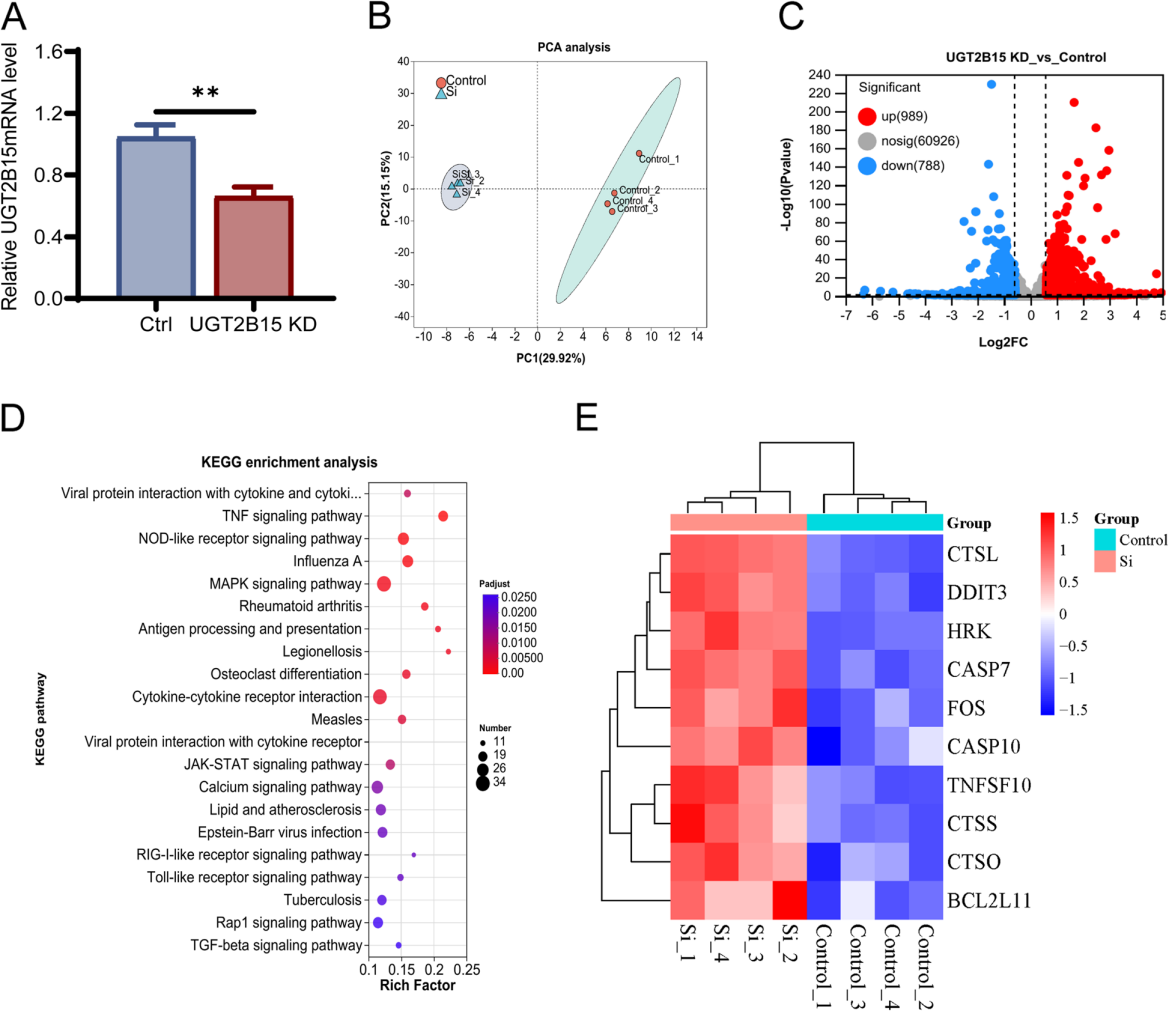
The effect of UGT2B15 knockdown on the transcriptome of KGN cells. **A** Validation of UGT2B15 gene expression in RNA-seq. **B** PCA analysis of all RNA-seq samples of two groups. **C** Volcano plot of RNA-seq results. **D** Performing KEGG enrichment analysis on differentially expressed genes. **E** Heat map of DEGs associated with apoptosis in two groups. *P* values were determined by one-way ANOVA with Tukey's multiple comparison post-hoc test. **P < 0.01

We analyzed UGT2B15 knockdown in KGN cells using RNA sequencing (RNA-seq), constructing eight libraries with four replicates in each. After processing, high-quality reads aligned to the human genome GRCh38 utilizing TopHat confirmed effective UGT2B15 knockdown (Fig. 2A). Principal component analysis (PCA) demonstrated clear separation between control and UGT2B15 knockdown groups (Fig. 2B), revealing 1777 differentially expressed genes (DEGs). A total of 989 upregulated and 788 downregulated DEGs were discovered using the criteria of fold change ≥ 1.5 or ≤ 0.5 and FDR < 0.05 (Fig. 2C). The results suggest that reducing UGT2B15 expression has a substantial impact on the expression levels of many genes. KEGG analysis indicated these DEGs are involved in the TNF signaling and apoptosis pathways (Fig. 2D and E).

**Fig. 3:**
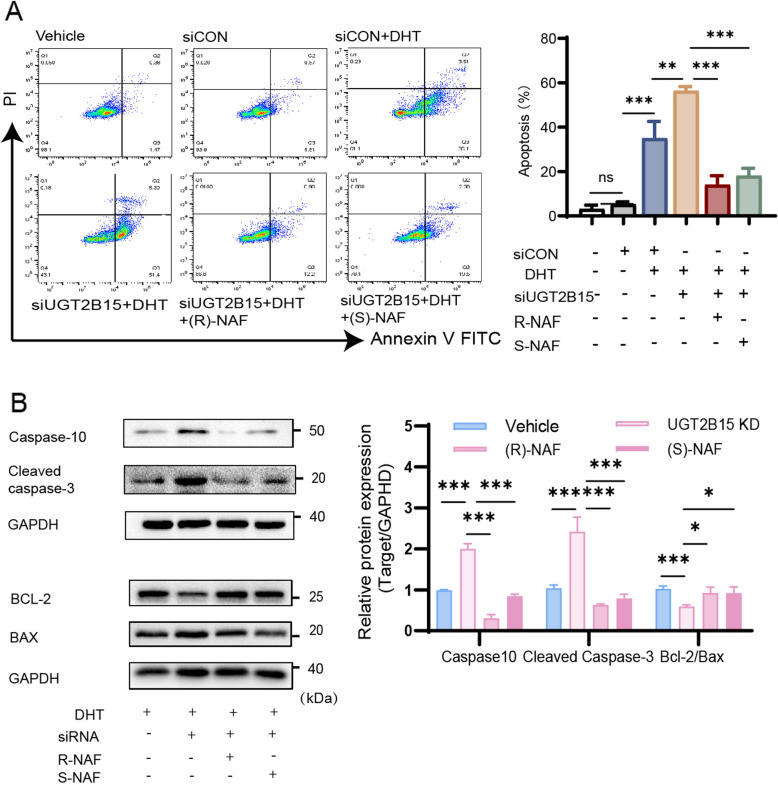
UGT2B15 ameliorated the apoptosis induced by androgen excess in KGN cells. **A** UGT2B15 KD and NAF enantiomers had contrasting effects on reducing the apoptosis of KGN cells caused by excessive DHT. **B** UGT2B15 KD and NAF enantiomers had contrasting effects on the modulation of Caspase10, Cleaved caspase3, Bcl-2/Bax gene expression induced by excessive DHT. All qPCR and Western blot results were standardized based on GAPDH levels and are presented in comparison to the control group. Values are expressed as mean ± SD (*n* = 3 per group). *P* values were determined by one-way ANOVA with Tukey's multiple comparison post-hoc test. *P* values less than 0.05, 0.01, and 0.001 are denoted as *, **, and ***, respectively, while ns indicates no significant distinction

Afterward, flow cytometry demonstrated that UGT2B15 knockdown increased apoptosis due to DHT accumulation, while *(R)*-NAF and *(S)*-NAF treatment reduced it, as illustrated in Fig. 3A. Moreover, increased levels of Caspase-10, Cleaved caspase-3, and decreased Bcl-2/Bax were observed in UGT2B15 knockdown cells, which reversed after exposure to *(R)*-NAF and *(S)*-NAF (Fig. 3B).

To summarize, the RNA-seq results indicated that UGT2B15 suppresses cell death in KGN cells, supported by further validation experiments using siRNA and *(R)*-NAF and *(S)*-NAF.

### *(R)*-NAF and *(S)*-NAF, as UGT2B15 inducers, improved PCOS mice phenomenon

**Fig. 4:**
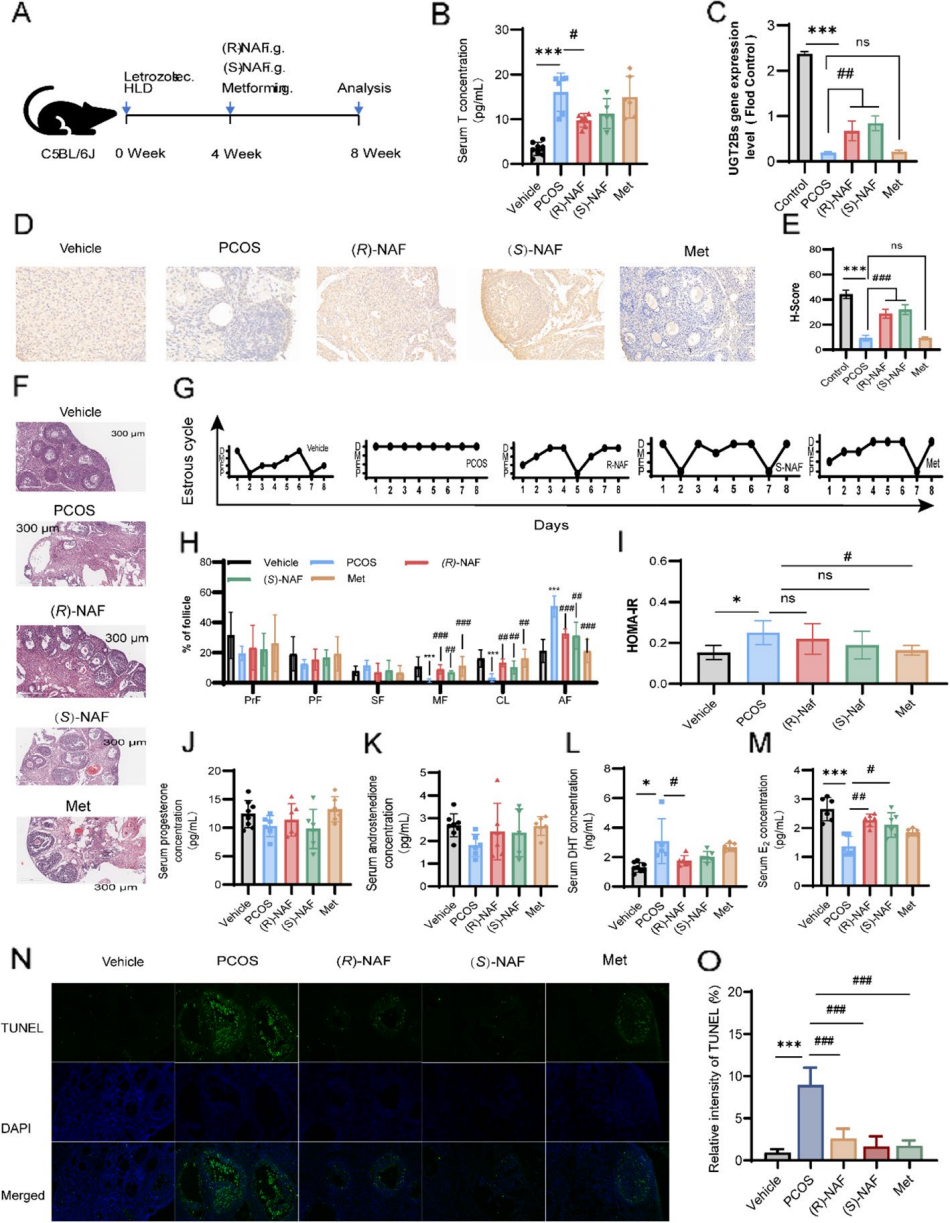
The impact of *(R)*-NAF and *(S)*-NAF on the reproductive and metabolic characteristics of PCOS mice caused by letrozole and high-fat diet (HFD) (*n* = 4–8). **A** Schematic diagram of the experimental protocols for *(R)/(S)*-NAF in treating letrozole with HFD-induced PCOS mice. **B** The serum levels of T. **C** The mRNA expression of UGT2Bs in the ovarian tissue of mice. **D** Immunohistochemical analysis of the protein expression level of UGT2Bs in ovarian tissues of each group, scale bar = 100 µm. **E** The histogram shows the H-score of UGT2Bs IHC in each group. **D** The mRNA expression of UGT2Bs in the ovarian tissue of mice. **F** Ovarian morphology was represented using hematoxylin and eosin staining, with a scale bar of 300 μm, showing primary follicles (triangle), secondary follicles (square), mature follicles (asterisk), cystic follicles or atretic follicles (asterisk), and corpus leteum (CL). **G** Representative of estrous cycles. **H** Percentages of each follicle type per ovary. Different stages of follicles include primordial follicles (PrF), primary follicles (PF), secondary follicles (SF), mature follicles (MF), atretic follicles (AF), and corpus leteum (CL). **I** Differential HOMA-IR of each group. **J**-**M** The serum levels of progesterone, androstenedione, DHT, and E2. **N** Representative images of ovarian stained by TUNEL assay, cell nuclei were stained by DAPI (blue), and apoptotic cells were stained with TUNEL (green). Scale bar = 100 μm. **O** Quantification of the proportion of ovarian apoptotic cells as detected by TUNEL assay in each group. The data is displayed as mean ± SD. Statistical analysis was performed using one-way ANOVA with Tukey’s post-hoc test for normally distributed data, or the Kruskal–Wallis test for non-normally distributed or unequal variance data. Significance levels: ^*^P < 0.05, ^**^P < 0.01, ^***^P < 0.001 (vs. vehicle group); ^#^*P* < 0.05, ^##^*P* < 0.01, ^###^*P* < 0.001 (vs. model group); ns, no significant difference (*P* > 0.05)

We assessed the characteristics of PCOS mice treated with *(R)*-NAF and *(S)*-NAF to study how repaired androgen metabolism affects the development of PCOS (Fig. 4A). Initially, the expression levels of UGT2Bs in mice were assessed using immunohistochemistry (IHC), showing a significant decrease in PCOS mice (Mean H-score 9.7%) compared to the vehicle group (Mean H-score 44.9%) (Fig. 4B). Compared to the vehicle group (Mean H-score 44.9%), UGT2Bs expression significantly decreased in the PCOS group (Mean H-score 9.7%). Treatment with *(R)*-NAF (Mean H-score 26.4%) and *(S)*-NAF (Mean H-score 36.5%) increased UGT2Bs expression, with similar patterns observed in mRNA levels (Fig. 4B-D). Consistent with findings in KGN cells, UGT2Bs expression was reduced in PCOS mice but increased after treatment with *(R)*-NAF and *(S)*-NAF.

Histomorphological examination of the ovaries demonstrated significant differences across the groups (Fig. 4E). Normal mice exhibited follicles at different developmental stages along with theca cells, granulosa cells (GCs), and corpus luteum (CL), showing no structural abnormalities or ovarian cysts. Conversely, the ovaries of the PCOS group showed a cystic appearance, thinner walls of follicles, and a reduced number of granulosa cell layers. Follicle growth analysis indicated fewer mature follicles and corpora lutea, but unchanged primordial, primary, and secondary follicles in PCOS mice (Fig. 4F). The administration of *(R)*-NAF, *(S)*-NAF, and metformin led to a significant enhancement in the quantities of mature follicles, corpora lutea, and granulosa cell layers compared to the PCOS group (Fig. 4E and F). PCOS mice had irregular estrous cycles after 4 weeks of letrozole administration with HFD, but *(R)*-NAF and *(S)*-NAF, and metformin partially restored the cyclicity (Fig. 4G).

A panel of serum hormone levels regulated by UGT2Bs showed that PCOS mice had higher T and DHT levels compared to the vehicle group, while *(R)*-NAF decreased T and DHT and increased E2 levels; *(S)*-NAF had similar but non-significant effects (Fig. 4I-M). Metformin did not display a trend in regulating T, DHT, and E_2_ serum levels. However, Metformin significantly improved the HOMA-IR score, while no significant effects were observed with *(R)*-NAF and *(S)*-NAF treatments (Fig. 4H).

Since we observed that the regulation of UGT2B15 may influence ovarian cell apoptosis in vitro, we next assessed the effect of *(R)*-NAF and *(S)*-NAF on ovarian cell apoptosis using a TUNEL assay. The results showed a significant increase in the number of TUNEL-positive cells in the ovaries of PCOS mice compared to the vehicle-treated mice (Fig. 4N and O). Interestingly, treatment with *(R)*-NAF and *(S)*-NAF significantly reduced the number of TUNEL-positive cells compared to the PCOS group. This suggests that while the PCOS model is associated with increased ovarian cell apoptosis, *(R)*-NAF and *(S)*-NAF treatment effectively mitigates apoptosis in ovarian cells.

In summary, *(R)*-NAF and *(S)*-NAF ameliorate PCOS by regulating UGT2Bs and reducing androgen accumulation, employing a mechanism distinct from that of Metformin.

### NR1H4 negatively regulated the UGT2B15 transcriptions in KGN cells

**Fig. 5:**
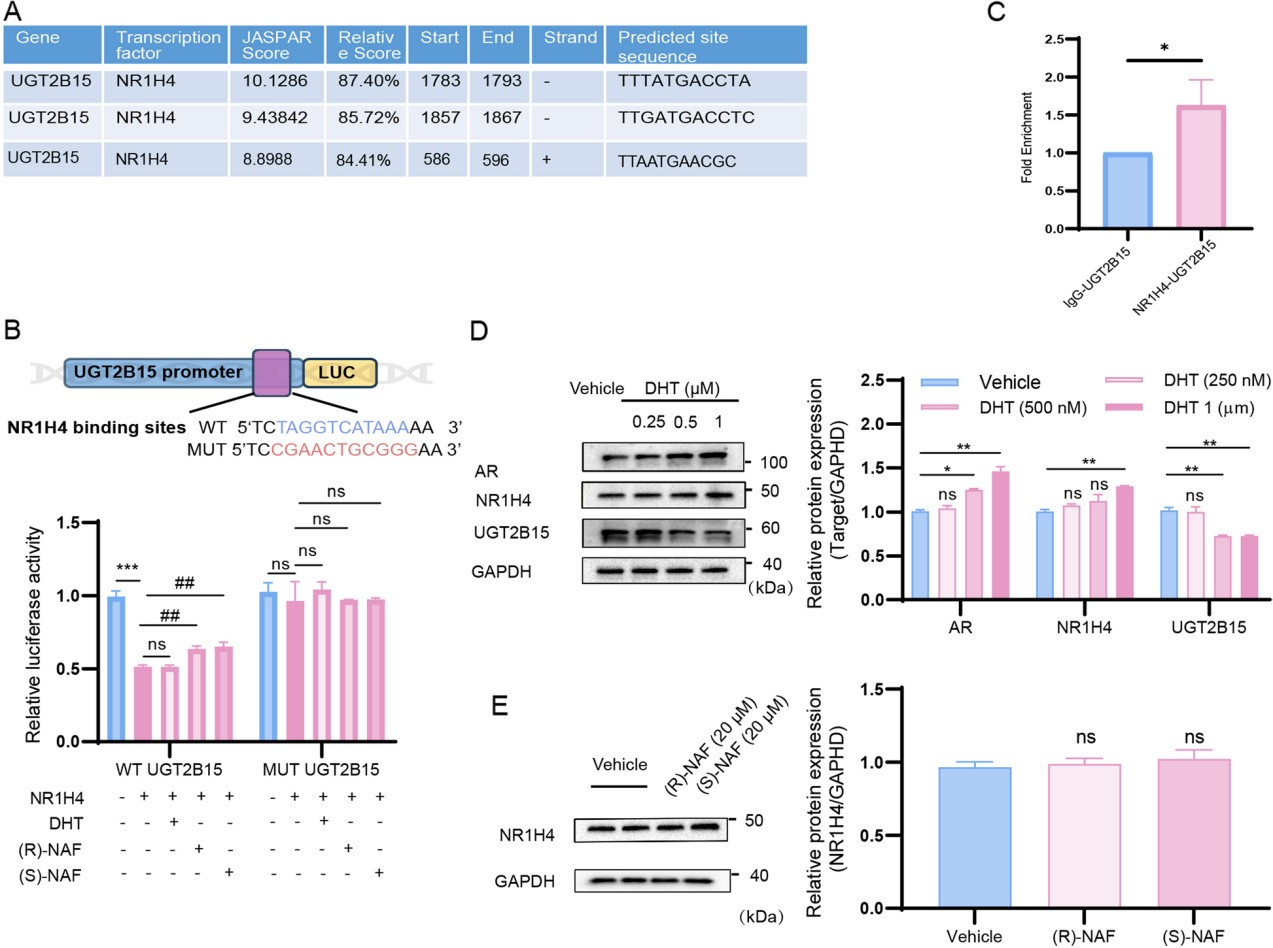
NR1H4 regulates UGT2B15 transcription and its interaction is influenced by DHT and NAF treatments. **A** JASPAR online analysis predicted NR1H4 binding sites in the promoter region of the human UGT2B15 gene (http://jaspar.genereg.net). **B** Dual-luciferase reporter assays were performed to validate the connection between UGT2B15 gene promoter regions and NR1H4, and the effects of DHT, *(R)*-NAF, and *(S)*-NAF on this process. **C** Chromatin immunoprecipitation (ChIP) study. **D** Effects of DHT on AR, NR1H4, and UGT2B15 protein levels in KGN cells. **E** Effects of *(R)*-NAF and *(S)*-NAF on NR1H4 protein levels in KGN cells. All qPCR and Western blot results were standardized based on GAPDH levels and are presented in comparison to the control group. Values are expressed as mean ± SD (*n* = 3–5 per group). *P* values were determined by one-way ANOVA with Tukey's multiple comparison post-hoc test. *P and ^#^*P* < 0.05, **P and ^##^*P* < 0.01, ***P and ^###^*P* < 0.001, ns means no significant difference

UGT gene transcription is mainly regulated by different tissue-specific and ligand-activated transcription factors (TFs). Previous studies on the transcriptional regulation of UGT2B15 have primarily focused on prostate cancer cells. These studies demonstrated, through luciferase and ChIP assays, that the androgen receptor (AR) binds to the *3’* promoter, thereby downregulating its transcription [36, 37]. Nuclear Receptor Subfamily 1 Group H Member 4 (NR1H4) has also been implicated in the transcriptional regulation of UGT2B15. Both natural (e.g., CDCA) and synthetic (e.g., GW4064) NR1H4 activators have been shown to suppress UGT2B15 expression and activity. However, direct evidence identifying the specific promoter elements mediating this regulation remains unidentified [38]. This study aims to elucidate the direct molecular mechanisms underlying NR1H4's regulation of UGT2B15 transcription in ovarian cells. Using the JASPAR database (http://jaspar.genereg.net), we identified potential NR1H4 binding regions in the UGT2B15 promoter (Fig. 5A). We assessed NR1H4's interaction with this promoter via luciferase reporter assays, constructing vectors with wild-type (WT-UGT2B15) or mutated (MUT-UGT2B15) NR1H4-binding sequences. Furthermore, we investigated the effects of DHT, *(R)*-NAF, and *(S)*-NAF on the interaction. Results showed a significant reduction in luciferase activity in the WT-UGT2B15 group when co-transfected with NR1H4, compared to the MUT-UGT2B15 group. This finding suggests that NR1H4 likely binds to the *UGT2B15* promoter, exerting a negative regulatory effect on its transcription. Notably, DHT did not significantly influence this process. Interestingly, treatment with NAF led to an increase in luciferase activity, implying that *(R)*-NAF and *(S)*-NAF may counteract the negative regulatory effect of NR1H4 on UGT2B15 expression (Fig. 5B). ChIP assays validated these binding sites, revealing the potential interaction between NR1H4 and the *UGT2B15* promoter in KGN cells (Fig. 5C). To further explore the effects of DHT and *(R)/(S)*-NAF on the interaction between NR1H4 and the UGT2B15 promoter, we analyzed their impact at the protein level. The results showed that increasing DHT concentrations led to a slight upregulation of NR1H4 protein levels, while UGT2B15 protein levels decreased significantly in a dose-dependent manner. Notably, the protein levels of the DHT receptor, AR, were markedly elevated with higher DHT concentrations. These findings, in conjunction with the luciferase assay results, suggest that DHT does not directly modulate the interaction between NR1H4 and the UGT2B15 promoter, nor does it directly influence NR1H4-mediated transcriptional regulation. Instead, DHT may regulate UGT2B15 transcription indirectly, potentially through AR or other upstream regulatory factors of UGT2B15.

Conversely, *(R)*-NAF and *(S)*-NAF did not significantly affect NR1H4 protein levels but appeared to directly interfere with the binding of NR1H4 to the UGT2B15 promoter, thereby mitigating NR1H4-mediated negative transcriptional regulation of UGT2B15. Further investigation is needed to clarify the mechanisms underlying the effects of *(R)*-NAF and *(S)*-NAF, including their specific binding interactions with NR1H4 and the UGT2B15 promoter region.

## Discussion

UGT enzymes facilitate glucuronidation for detoxifying substances, with 22 human subtypes in four families: UGT1, UGT2, UGT3, and UGT8. The roles of members in the UGT1A and UGT2B families have been well-documented [39]. UGT2B family, particularly UGT2B15, is the principal UGT enzyme responsible for local androgen glucuronidation in steroid-target tissues influencing androgen response and regulating steroid levels [26, 27, 39]. Targeting UGT2B15 may provide a therapeutic approach for androgen-related diseases like prostate and breast cancer [28, 29, 39]. Past research on UGT2B15 in ovary tissue and cells of cynomolgus monkeys revealed that UGT2B proteins are present in the cytoplasm of thecal and granulosa cells in developing follicles and coincide with AR in the corresponding cell types of the ovary. It has been indicated that UGT2B15 and AR co-regulated the levels and activities of androgens in the ovary [21]. Furthermore, in an environmental endocrine disruptors (EEDs) study, the homozygous variants of *UGT2B15*^*D85Y*^ were linked to the capacity of the excretion of androgen and the clearance of EEDs [22]. All of the above indicated the potential therapeutic role of UGT2B15 in PCOS treatment.

The growth of granulosa cells is crucial for follicular development, as it influences steroid hormone secretion and communication with oocytes. Dysfunction in granulosa cells affects follicle quality [13]. Androgens once thought to mainly impact males, are crucial for regulating ovarian steroid hormones in females, with excess linked to follicular dysplasia and PCOS [40]. However, the exact mechanisms remain to be fully understood. In this study, we first identified the expression of the UGT2B family in KGN cell lines, revealing that excessive DHT reduces UGT2B15 levels. RNA-seq data and verification experiments demonstrated that UGT2B15 significantly inhibited DHT accumulation and protected against apoptosis of KGN cells, suggesting a potential role for UGT2B15 in androgen excess-related female diseases.

Numerous research studies have explored how UGT2B15 expression is controlled by long non-coding RNAs (lncRNAs), microRNAs (miRNAs), and DNA methylation, but research on chemical compounds affecting UGT2B15 is limited [39]. Naftopidil (NAF), an α1D/1A-adrenoceptor antagonist for LUTS in BPH, induces smooth muscle relaxation [41]. Our prior research indicated that NAF enantiomers could serve as novel and effective regulators of UGT2B15, enhancing the elimination of DHT and promoting apoptosis in BPH-1 cells [30]. This study aims to evaluate the pharmacological effects of *(R)*-NAF and *(S)*-NAF in vitro and in vivo tests. In KGN cells, UGT2B15 knockdown followed by rescue experiments with *(R)*-NAF and *(S)*-NAF confirmed that increased UGT2B15 levels suppressed apoptosis by reducing androgen accumulation. In a PCOS mouse model, both *(R)*-NAF and *(S)*-NAF upregulated *Ugt2b* gene expression, alleviating hyperandrogenemia, ovulatory dysfunction, and polycystic ovarian morphology. Although UGT enzymes in mice function similarly to human UGT2B15, identifying orthologues across species remains challenging due to high sequence similarity (> 70%) among UGT2B family members. In mice, *Ugt2b* genes are located on chromosome 14p21, with isoforms such as *Ugt2b1*, *Ugt2b5*, and *Ugt2b37* playing crucial roles in androgen glucuronidation [42, 43]. Consequently, we assessed the expression of total UGT2Bs using an anti-UGT2B antibody in immunohistochemistry (IHC), confirming that *(R)*-NAF and *(S)*-NAF regulate UGT2Bs and positively impact PCOS in mice. Moreover, as an approved drug with established clinical use, NAF is safer and more cost-effective than other new drug entities. Further studies should clarify the mechanisms and impacts of NAF enantiomers on UGT2B15 in different androgen-responsive cells and animal models.

The expression profiles of UGTs across different tissues have been extensively characterized in numerous studies. Various ligand-activated TFs that are activated by ligands, particularly those in the nuclear receptor superfamily like the AR, NR1H4, estrogen receptor alpha (ERα), constitutive androstane receptor (CAR), vitamin D receptor (VDR), and glucocorticoid receptor (GR) have been demonstrated to control UGT genes through either activation or repression [44, 45]. UGT2B15 is broadly expressed in tissues sensitive to sex hormones, including the testis, breast, uterus, prostate, and ovary, suggesting its pivotal role in local hormone metabolism [39]. Prior research in multiple ER-positive breast cancer cell lines has shown that FOXA1, functioning as a pioneering transcription factor, aids in opening chromatin and then attracting AR or ER to nearby promoter regions of UGT2B15 genes [46, 47]. Contrary to the stimulation observed with estrogens and androgens in breast cancer cells, DHT and synthetic androgens (R1881) inhibit UGT2B15 expression and activity in LNCaP cells [48]. ChIP assays confirm AR binding to UGT2B15 promoters, but the mechanism of androgen suppression in prostate cancer remains unclear [36]. Turning to NR1H4, both natural and synthetic NR1H4 activators inhibit UGT2B15 in LNCaP cells, although there is limited direct binding evidence [38]. This study confirmed the direct binding between NR1H4 and the UGT2B15 promoter through luciferase and ChIP assays. This study confirmed the direct binding of NR1H4 to the UGT2B15 promoter via luciferase and ChIP assays. Protein-level analysis indicated that while DHT slightly upregulated NR1H4 expression, it did not directly affect its interaction with the UGT2B15 promoter, suggesting alternative regulatory mechanisms involving AR or other upstream factors. In contrast, *(R)*-NAF and *(S)*-NAF disrupted NR1H4 binding to the promoter without altering its protein levels, indicating direct interference with NR1H4-mediated repression. These findings highlight the complexity of UGT2B15 regulation and suggest a potential role for NAF in modulating NR1H4 activity, warranting further investigation into its underlying mechanisms and therapeutic implications.

## Conclusion

This study reveals UGT2B15's potential as a therapeutic target for PCOS by enhancing androgen metabolism and protecting ovarian cells. The discovery of *(R)*-NAF and *(S)*-NAF as regulators of UGT2B15 expression implies the potential therapeutic compounds for PCOS treatment by modulating androgen metabolism. Additionally, NR1H4 negatively regulates UGT2B15 transcription in KGN cells. *(R)*-NAF and *(S)*-NAF may exert their regulatory effects on UGT2B15 by interfering with the interaction between NR1H4 and the UGT2B15 promoter. Future research should aim to elucidate the molecular mechanisms governing the regulation of UGT2B15 by new compounds and NR1H4 regulators and explore the therapeutic potential of targeting this pathway in PCOS treatment.

## Data Availability

No datasets were generated or analysed during the current study.
